# Adequação da oferta de procedimentos para a detecção precoce do câncer de mama no Sistema Único de Saúde: um estudo transversal, Brasil e regiões, 2019

**DOI:** 10.1590/0102-311XPT139723

**Published:** 2024-05-20

**Authors:** Maria Beatriz Kneipp Dias, Mônica de Assis, Renata Oliveira Maciel dos Santos, Caroline Madalena Ribeiro, Arn Migowski, Jeane Glaucia Tomazelli

**Affiliations:** 1 Instituto Nacional de Câncer, Rio de Janeiro, Brasil.; 2 Instituto Nacional de Cardiologia, Rio de Janeiro, Brasil.

**Keywords:** Early Detection of Cancer, Breast Neoplasms, Health Planning, Unified Health System, Health Services Programming, Detecção Precoce do Câncer, Neoplasias da Mama, Planejamento em Saúde, Sistema Único de Saúde, Programação de Serviços de Saúde, Detección Precoz del Cáncer, Neoplasias de la Mama, Planificación en Salud, Sistema Único de Salud, Programación de Servicios de Salud

## Abstract

A detecção precoce é uma das estratégias para o controle do câncer de mama e, para tanto, é fundamental garantir o acesso à investigação dos casos suspeitos para continuidade do cuidado e tratamento oportuno. Este estudo tem por objetivo estimar a necessidade de procedimentos para detecção precoce dessa neoplasia e avaliar a sua adequação no atendimento às mulheres rastreadas e sintomáticas no Sistema Único de Saúde (SUS), no ano de 2019. Foi realizado um estudo descritivo transversal para analisar a oferta de exames de detecção precoce do câncer de mama, comparando a necessidade estimada com os procedimentos realizados no SUS. Foram utilizados os parâmetros disponibilizados pelo Instituto Nacional de Câncer para estimar a população e a necessidade de exames para a detecção precoce. No Sistema de Informações Ambulatoriais do SUS, obteve-se o número de procedimentos realizados em 2019. Observou-se um déficit de mamografias de rastreamento no país (-45,1%), variando entre -31,4% na Região Sul a -70,5% na Região Norte. Se a oferta desse exame fosse direcionada para a população-alvo do rastreamento, o déficit no país reduziria para -14,8% e haveria sobreoferta no Sul (6,2%). Os procedimentos de investigação diagnóstica apresentaram variações entre as regiões, com maiores déficits de punção por agulha grossa (-90,8%) e biópsia/exérese de nódulo da mama (-80,6%) observados no Centro-oeste, e o maior déficit de exames anatomopatológicos no Norte (-88,5%). A comparação entre a produção e a necessidade de procedimentos para detecção precoce do câncer de mama no Brasil identificou déficits e inadequações que devem ser melhor conhecidos e equacionados em nível estadual e municipal.

## Abstract (en)

Early detection is a major strategy in breast cancer control and, for this reason, it is important to ensure access to investigation of suspected cases for care continuity and timely treatment. This study aimed to estimate the need for procedures of breast cancer early detection and assess their adequacy for providing care to screened and symptomatic women in the Brazilian Unified National Health System (SUS) in 2019. A descriptive cross-sectional study was conducted to analyze the provision of tests for breast cancer early detection, comparing the estimated need with the procedures performed in the SUS. Parameters provided by the Brazilian National Cancer Institute were used to estimate the population and the need for early detection tests. The number of procedures performed in 2019 was obtained from the Outpatient Information System of the SUS. A deficit in screening mammograms was observed in the country (-45.1%), ranging from -31.4% in the South Region to -70.5 % in the North Region. If this test was offered to the target population, the deficit in the country would reduce to -14.8% and there would be an oversupply in the South Region (6.2%). Diagnostic investigation procedures varied between the regions, with higher deficits in coarse needle biopsy (-90.8%) and breast lump biopsy/excision (-80.6%) observed in the Central-West Region, and the highest deficit in anatomopathological exams in the North Region (-88.5%). The comparison between the production and need for procedures of breast cancer early detection in Brazil and its regions identified deficits and inadequacies that must be better understood and addressed at the state and municipal levels.

## Resumen

La detección temprana es una de las estrategias para el control del cáncer de mama y, para ello, es fundamental garantizar el acceso a la investigación de los casos sospechosos para la continuidad del cuidado y el tratamiento oportuno. El presente estudio tiene como objetivo estimar la necesidad de procedimientos para la detección temprana de esta neoplasia y evaluar su adecuación en la atención a las mujeres rastreadas y sintomáticas en el Sistema Único de Salud (SUS) brasileño, en el año 2019. Se realizó un estudio descriptivo transversal para analizar la oferta de pruebas para la detección temprana del cáncer de mama, comparando la necesidad estimada con los procedimientos realizados en el SUS. Se utilizaron los parámetros proporcionados por el Instituto Nacional del Cáncer para estimar la población y la necesidad de pruebas para la detección temprana. El número de procedimientos realizados en el 2019 se obtuvo del Sistema de Información Ambulatoria del SUS. Se observó un déficit de mamografías de tamizaje en el país (-45,1%), oscilando entre el -31,4% en la Región Sur y el -70,5% en la Región Norte. Si la oferta de esta prueba se dirigiera a la población objetivo del rastreo, el déficit en el país se reduciría al -14,8% y habría una sobreoferta en el Sur (6,2%). Los procedimientos de investigación diagnóstica presentaron variaciones entre regiones, observándose mayores déficits en punción con aguja gruesa (-90,8%) y biopsia/escisión de nódulo mamario (-80,6%) en el Centro-Oeste, y el mayor déficit de pruebas anatomopatológicas en el Norte (-88,5%). La comparación entre la producción y la necesidad de procedimientos para la detección temprana del cáncer de mama en Brasil y en las regiones identificó déficits e insuficiencias que deben ser mejor conocidos y abordados a nivel estatal y municipal.

## Introdução

O câncer de mama é a neoplasia maligna que mais acomete mulheres em todo o mundo, representando 11,7% dos cânceres em mulheres, excluídos os de pele não melanoma ^1^. Em 2020, os casos novos de câncer de mama superaram os de câncer de pulmão, além de representarem a principal causa de morte por câncer em mulheres. Esse cenário afeta, sobretudo, os países de média e baixa renda em que o acesso ao diagnóstico e ao tratamento são limitados por falta de estrutura da rede assistencial. A sobrevida em câncer de mama atualmente varia de 90% em países de alta renda a 66% na Índia e 40% na África ^2^.

A detecção precoce do câncer de mama é influenciada diretamente pela organização da rede de serviços de saúde e tem impacto no prognóstico da doença ^3^, cabendo à gestão planejar e implementar ações para estabelecer o cuidado integral. O diagnóstico precoce (abordagem oportuna de mulheres com sinais e sintomas suspeitos) e o rastreamento (exames periódicos em faixa etária de maior risco de adoecimento) são as estratégias de detecção precoce a serem organizadas. A primeira dirige-se ao conjunto da população feminina e a segunda às mulheres na faixa etária de 50 a 69 anos, recomendada nas diretrizes brasileiras ^4^ como a que melhor se beneficia do rastreamento mamográfico, a exemplo do que ocorre em programas organizados de rastreamento no contexto internacional ^5^, ainda que permaneça a polêmica em torno de possível antecipação da idade do rastreio para 40 anos ^6^.

Para assegurar as ações de detecção precoce na linha de cuidado do câncer de mama, é necessário garantir o acesso da população à avaliação e à investigação dos casos suspeitos, assegurando a continuidade do cuidado e o tratamento em tempo oportuno. Mulheres assintomáticas que apresentam alterações na mamografia de rastreamento devem realizar exames de investigação diagnóstica para confirmar ou descartar o câncer. Mulheres com sinais ou sintomas suspeitos de câncer de mama necessitam de rápida avaliação e exames de investigação diagnóstica, conforme a idade e o tipo de alteração apresentada ^7^. Esse grupo deve ser priorizado em função do maior risco de confirmação de câncer ^4^. As dificuldades de acesso à investigação diagnóstica e ao tratamento resultam em tempos longos do percurso assistencial das mulheres ^8^^,^^9^ e, consequentemente, em proporções elevadas de diagnóstico em estágios avançados ^10^^,^^11^.

Historicamente, a programação em saúde no Brasil foi baseada em série histórica dos exames realizados, porém, mais recentemente, busca-se uma melhor aproximação com as necessidades de saúde da população por meio do conhecimento da situação de saúde, organização da rede de serviços e estabelecimento de critérios e parâmetros ^12^.

Para o controle do câncer de mama, foram recém-publicados os parâmetros de programação da oferta de procedimentos para atender as demandas das mulheres assintomáticas ^13^ e sintomáticas (diagnóstico precoce) ^14^. Tais parâmetros possibilitam que estados e municípios estimem a necessidade e planejem as ações, de forma integrada, em cada território. Permitem avaliar o déficit ou a sobreoferta de procedimentos ao comparar a necessidade com a produção registrada nos sistemas de informação ^15^^,^^16^.

Além do acesso ao tratamento e às diversas modalidades terapêuticas, a oferta suficiente e qualificada de serviços para o rastreamento e o diagnóstico precoce do câncer de mama é um componente essencial para o alcance da meta de redução de 10% na mortalidade prematura por esse tipo de câncer, assumida no plano de enfrentamento das doenças crônicas no Brasil ^17^.

Tendo em vista a urgência de se organizar efetivamente a linha de cuidado do câncer de mama no país, o objetivo deste estudo é estimar a necessidade de procedimentos para a detecção precoce dessa neoplasia e avaliar a adequação dos procedimentos realizados no atendimento às mulheres rastreadas e sintomáticas no Sistema Único de Saúde (SUS) no ano de 2019.

## Métodos

Estudo descritivo transversal sobre a necessidade estimada e os procedimentos realizados para análise da oferta de exames na detecção precoce do câncer de mama no SUS.

A estimativa de necessidade de procedimentos foi calculada utilizando método descrito em publicações do Ministério da Saúde que estabelecem parâmetros para a programação dos procedimentos da linha de cuidado do rastreamento ^13^ e diagnóstico precoce do câncer de mama ^14^.

O SUS é um sistema de saúde universal que contempla a toda a população brasileira, sendo que aproximadamente 75% das pessoas dependem exclusivamente dele ^18^. Como há procedimentos que são realizados por desembolso direto ou por meio de plano/seguro de saúde privados, cujos dados não estão disponíveis para análise, a cobertura da saúde suplementar foi subtraída do cálculo da população feminina para evitar a superestimação da necessidade de procedimentos.

Para obter a população feminina usuária do SUS, aplicou-se, então, a cobertura da população feminina da saúde suplementar de 2019 ^18^ na população feminina total desse mesmo ano, definindo-se o número de mulheres cobertas pela saúde suplementar. Posteriormente, excluiu-se esse número da população feminina total estimada pelo Instituto Brasileiro de Geografia e Estatística (IBGE) para 2019, utilizando-se os cálculos a seguir:

Número de mulheres cobertas pela saúde suplementar:

númerototaldemulheresestimadopeloIBGEde2019×coberturadapopulaçãofemininadasaúdesuplementarde2019

População feminina total usuária do SUS:

númerototaldemulheresestimadopeloIBGEem2019-númerodemulherescobertaspelasaúdesuplementarem2019

A população feminina total usuária do SUS foi utilizada como base para a definição do número previsto de mulheres sintomáticas.

O número de mulheres sintomáticas para câncer de mama foi calculado para o Brasil e as regiões aplicando os parâmetros técnicos para detecção precoce do câncer de mama ^14^, que utilizam a combinação de dados dos Registros Hospitalares de Câncer e das estimativas nacionais de casos novos de câncer. Para a estimativa de mulheres sintomáticas do Brasil, os parâmetros de 0,048% e 0,522% foram aplicados à população feminina abaixo dos 30 anos e com 30 anos ou mais, respectivamente. Para a análise dos dados regionais, foram calculados parâmetros específicos, a partir das estimativas de incidência de cada região para o ano de 2020 (Tabela 1). Foram utilizadas as estimativas de incidência do ano de 2020 por serem a melhor correspondência temporal para a análise proposta.

**Tabela 1:** População feminina, cobertura de saúde suplementar, cobertura de mamografia e estimativa de incidência de câncer de mama. Brasil e regiões, 2019.

Região	População feminina em 2019 *		Cobertura de saúde suplementar em 2019 **		Cobertura de rastreamento da PNS 2019 *** - 50 a 69 anos (%)	Taxa bruta de incidência de câncer de mama ^#^
	Todas as mulheres	Mulheres de 50 a 69 anos	População feminina	Mulheres de 50 a 69 anos		
Norte	9.165.776	1.238.490	11,0	15,8	43,2	21,34
Nordeste	29.390.778	5.157.191	12,9	15,4	49,5	44,29
Sudeste	45.326.845	9.766.094	36,4	39,7	65,2	81,06
Sul	15.281.620	3.404.411	25,7	27,0	58,8	71,16
Centro-oeste	8.221.848	1.500.974	22,8	27,3	56,6	45,24
Brasil	107.386.867	21.067.160	25,2	29,5	58,3	61,61

PNS: *Pesquisa Nacional de Saúde*.

* De acordo com dados do Departamento de Informática do SUS ^51^;

** De acordo com dados da Agência Nacional de Saúde Suplementar ^18^;

*** De acordo com dados da PNS ^19^;

# Estimativa de incidência por 100 mil mulheres ^22^.

Para o cálculo do número de mulheres rastreadas no Brasil e suas regiões, foram utilizados os percentuais de cobertura de mamografia em mulheres de 50 a 69 anos, oriundos da última edição da *Pesquisa Nacional de Saúde* (PNS) ^19^. Assumiu-se que esses patamares de cobertura, embora menores do que os 70% estabelecido como meta para o Brasil até 2030 ^17^, aproximam-se mais da real cobertura do rastreamento mamográfico. Desse total, foi subtraído o número de mulheres com cobertura de saúde suplementar do mesmo grupo etário, para o país e as regiões, conforme a memória de cálculo apresentada a seguir:

População feminina de 50 a 69 anos rastreada:

númerototaldemulheresnafaixaetáriade50a69anosestimadapeloIBGEem2019×coberturademamografiadaPNSde2019

População feminina de 50 a 69 anos rastreada usuárias do SUS:

populaçãofemininade50a69anosrastreadaem2019-coberturafemininanafaixaetáriade50a69anosnasaúdesuplementarem2019

Os valores de referência da população feminina, cobertura de saúde suplementar, cobertura de mamografia e incidência de câncer de mama utilizados para o cálculo das estimativas de necessidade de procedimentos são apresentados na Tabela 1.

Após definição da população rastreada e sintomática foram aplicados os parâmetros ^14^ para estimar o número de procedimentos necessários no atendimento às ações de detecção precoce do câncer de mama para o país e regiões, no ano de 2019.

Do Sistema de Informações Ambulatoriais do SUS (SIA/SUS), foram obtidos os procedimentos de rastreamento e investigação diagnóstica do câncer de mama realizados na população feminina no ano de 2019. Os procedimentos, definidos no Sistema de Gerenciamento da Tabela de Procedimentos, Medicamentos e OPM do SUS (SIGTAP) ^20^, foram:

Mamografia de rastreamento (02.04.03.018-8);

Mamografia (02.04.03.003-0), que corresponde à mamografia diagnóstica;

Ultrassonografia mamária (02.05.02.009-7);

Punção aspirativa por agulha grossa (PAG) (02.01.01.060-7), que corresponde à “*core*” biópsia;

Biópsia/exérese de nódulo de mama (02.01.01.056-9), que corresponde à “biópsia cirúrgica”;

Exame citopatológico de mama (02.0301.004-3);

Exame anatomopatológico de mama - biópsia (02.03.02.006-5).

Com exceção da mamografia de rastreamento, indicada para mulheres assintomáticas, todos os demais procedimentos são indicados para a investigação diagnóstica das alterações mamárias suspeitas, oriundas ou não do rastreamento ^13^^,^^14^.

Os procedimentos foram agrupados por região de residência das mulheres, a fim de aferir as possíveis variações internas na organização da rede assistencial do SUS.

A adequação entre o número de exames necessários e o quantitativo de procedimentos realizados em 2019 foi avaliada para Brasil e regiões por meio da comparação entre exames realizados e registrados no SIA/SUS e os totais estimados para cada procedimento. A avaliação indicou déficit, quando o resultado foi um valor negativo, ou sobreoferta, quando positivo, conforme o método de cálculo a seguir:

Percentual de déficit / sobreoferta:

$$\frac{(NúmerodedeterminadoprocedimentoregistradonoSIA\text{/}SUS-númeroestimadodesteprocedimento)\times 100}{NúmerodedeterminadoprocedimentoregistradonoSIA\text{/}SUS}$$

### Aspectos éticos

Conforme a *Resolução nº 510*, de 7 de abril de 2016, do Conselho Nacional de Saúde (CNS) ^21^, o projeto deste estudo não foi submetido à apreciação de um comitê de ética em pesquisa por utilizar exclusivamente dados secundários, de acesso público e sem a possibilidade de identificação dos indivíduos submetidos aos procedimentos de rastreamento e diagnóstico analisados.

## Resultados

A população estimada de mulheres sintomáticas para câncer de mama no país foi 457.855 mulheres, sendo 38.556 abaixo de 30 anos e 419.298 com 30 anos ou mais. Os menores percentuais de população feminina sintomática foram observados na Região Norte (0,017% e 0,181%), enquanto os mais elevados foram os da Região Sudeste (0,063% e 0,686%), nos grupos etários abaixo de 30 anos e com 30 anos ou mais, respectivamente (Tabela 2).

**Tabela 2:** Percentual e número estimado de mulheres sintomáticas, número estimado de mulheres rastreadas e estimativa de procedimentos para o rastreamento e investigação diagnóstica do câncer de mama no Sistema Único de Saúde (SUS). Brasil e regiões, 2019.

Parâmetros populacionais e de procedimentos				Brasil		Norte		Nordeste		Sudeste		Sul		Centro-oeste	
				n		n		n		n		n		n	
População feminina usuária do SUS				80.325.377		8.157.541		25.599.368		28.827.873		11.354.244		6.347.267	
				%	n	%	n	%	n	%	n	%	n	%	n
Parâmetro e população feminina sintomática		< 30 anos		0,048	38.556	0,017	1.387	0,034	8.704	0,063	18.162	0,055	6.245	0,035	2.222
		> 30 anos		0,522	419.298	0,181	14.765	0,375	95.998	0,686	197.759	0,603	68.466	0,383	24.310
Procedimentos		Parâmetros de diagnóstico (%)		Necessidade estimada de procedimentos para população sintomática											
		< 30 anos	> 30 anos	n		n		n		n		n		n	
Mamografia		28,15	147,10	627.642		22.110		143.663		296.016		102.472		36.385	
Ultrassonografia mamária bilateral		278,37	207,03	975.402		34.429		222.973		459.977		159.129		56.513	
Punção aspirativa por agulha grossa (PAG)		18,07	17,74	81.351		2.870		18.603		38.364		13.274		4.714	
Biópsia/Exérese de nódulo de mama		13,03	6,16	30.853		1.090		7.048		14.548		5.031		1.787	
Exame anatomopatológico de mama - biópsia		31,10	23,90	112.203		3.960		25.650		52.913		18.306		6.501	
Exame citopatológico de mama		3,44	2,50	11.809		417		2.699		5.569		1.926		684	
				n		n		n		n		n		n	
Estimativa da população feminina de 50 a 69 anos rastreada				12.282.154		535.028		2.552.810		6.367.493		2.001.794		849.551	
População feminina de 50 a 69 anos rastreada, usuárias do SUS				8.658.919		450.493		2.159.677		3.839.598		1.461.309		617.624	
Procedimentos		Parâmetros de rastreamento (%)		Necessidade estimada de procedimentos para população assintomática											
				n		n		n		n		n		n	
Mamografia bilateral para rastreamento		50,00		4.329.459		225.247		1.079.838		1.919.799		730.655		308.812	
Mamografia		2,90		251.109		13.064		62.631		111.348		42.378		17.911	
Ultrassonografia mamária bilateral		3,50		303.062		15.767		75.589		134.386		51.146		21.617	
Punção aspirativa por agulha grossa (PAG)		0,73		63.210		3.289		15.766		28.029		10.668		4.509	
Biópsia/Exérese de nódulo de mama		0,11		9.525		496		2.376		4.224		1.607		679	
Exame anatomopatológico de mama - biópsia		0,84		72.735		3.784		18.141		32.253		12.275		5.188	

Fonte: Departamento de Informática do SUS ^51^; Agência Nacional de Saúde Suplementar ^18^; Instituto Brasileiro de Geografia e Estatística ^19^; Instituto Nacional de Câncer ^14^.

Considerando a cobertura estimada pela PNS de 2019, cerca de 12 milhões de mulheres na faixa etária de 50 a 69 anos fizeram mamografia nos últimos dois anos no país, das quais 8,7 milhões foram provavelmente atendidas no SUS. Com base nas coberturas regionais, o número estimado de mulheres assintomáticas rastreadas no SUS variou de 450.493 mulheres na Região Norte a aproximadamente 3,8 milhões na Região Sudeste.

Ainda na Tabela 2, são apresentados os parâmetros de estimativas de procedimentos para rastreamento e diagnóstico precoce de câncer de mama e o quantitativo de exames necessários, considerando a população sintomática e de rastreamento usuárias do SUS, para o Brasil e as regiões.

Para mulheres sintomáticas, estimou-se a necessidade de cerca de 627 mil mamografias diagnósticas, 975 mil ultrassonografias mamárias, 81 mil PAGs, 30 mil exéreses de nódulos mamários, 112 mil exames anatomopatológicos-biópsias e, aproximadamente, 12 mil exames citopatológicos de mama, com variações importantes entre as regiões. Enquanto o Sudeste e Nordeste têm necessidade estimada de exames citopatológicos de mama próxima de 3 e 6 mil exames, respectivamente, nas regiões Norte e Centro-oeste essa estimativa é inferior a 1.000 exames.

Para mulheres assintomáticas, na faixa etária de 50 a 69 anos, estimou-se para o país a necessidade de 4,3 milhões de mamografias de rastreamento, 251 mil mamografias diagnósticas, 303 mil ultrassonografias mamárias, 63 mil PAGs, 9 mil exéreses de nódulo de mama e 72 mil exames anatomopatológicos-biópsia (Tabela 2).

Na Tabela 3, são apresentados o número de procedimentos registrados no SIA/SUS e o total de procedimentos estimados para mulheres sintomáticas e assintomáticas.

**Tabela 3:** Número de procedimentos estimados e procedimentos realizados para a detecção precoce do câncer de mama no Sistema Único de Saúde (SUS). Brasil e regiões, 2019.

Procedimentos	Necessidade/Registrado	Total de procedimentos estimados e registrados no SIA/SUS (n)					
		Brasil	Norte	Nordeste	Sudeste	Sul	Centro-oeste
Mamografia bilateral para rastreamento	Necessidade	4.329.459	225.247	1.079.838	1.919.799	730.655	308.812
	Registrado no SIA/SUS	3.688.378	116.222	886.413	1.733.154	775.864	176.725
	Registrado no SIA/SUS entre 50 e 69 anos	2.376.526	66.364	587.317	1.115.738	501.442	105.665
Mamografia	Necessidade	878.750	35.174	206.293	407.365	144.850	54.297
	Registrado SIA/SUS	208.965	5.135	28.557	119.976	44.858	10.439
Ultrassonografia mamária bilateral	Necessidade	1.278.465	50.196	298.561	594.363	210.275	78.130
	Registrado SIA/SUS	1.303.817	61.898	378.550	597.623	193.405	72.341
Punção aspirativa por agulha grossa (PAG)	Necessidade	144.561	6.159	34.368	66.393	23.942	9.223
	Registrado SIA/SUS	33.498	1.471	10.406	15.674	5.097	850
Biópsia/Exérese de nódulo de mama	Necessidade	40.377	1.586	9.423	18.772	6.639	2.466
	Registrado SIA/SUS	11.503	1.110	2.621	5.560	1.733	479
Exame anatomopatológico de mama - biópsia	Necessidade	184.398	7.744	43.792	85.165	30.581	11.689
	Registrado SIA/SUS	42.849	889	11.625	20.819	6.145	3.371
Exame citopatológico de mama	Necessidade	11.809	417	2.699	5.569	1.926	684
	Registrado SIA/SUS	20.005	792	8.325	6.382	2.331	2.175

SIA/SUS: Sistema de Informações Ambulatoriais do SUS.

Fonte: Departamento de Informática do SUS ^52^.

No ano de 2019, aproximadamente 3,7 milhões de mamografias de rastreamento foram registradas no SIA/SUS, das quais 2.376.526 foram realizadas em mulheres na faixa etária de 50 a 69 anos.

**Figura 1:**
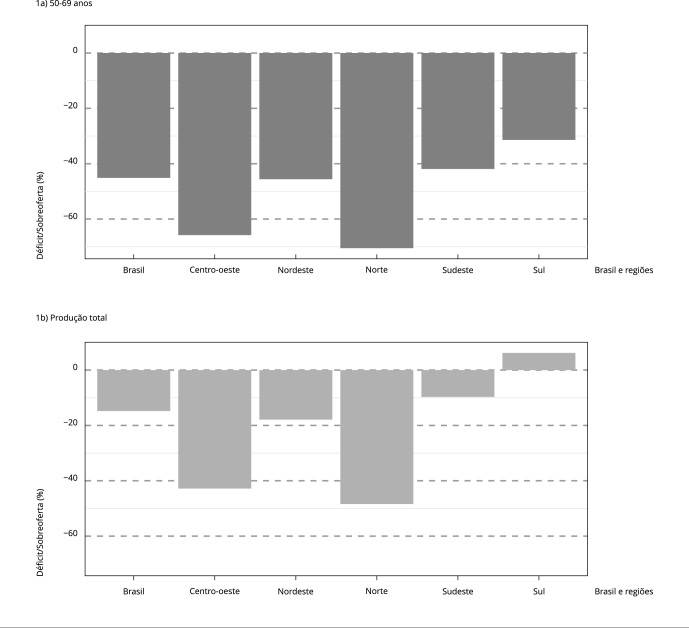
Déficit e sobreoferta de mamografia de rastreamento em mulheres de 50 a 69 anos, estimados com base na realização de mamografia de rastreamento no Sistema Único de Saúde (SUS) *. Brasil e regiões, 2019.

Considerando a necessidade estimada para rastrear a população-alvo no mesmo ano, no SUS, observou-se um déficit de mamografias de rastreamento no país (-45,1%). Entre as regiões, o déficit variou de -31,4% no Sul a -70,5% no Norte (Figura 1).

* Considerados os patamares de cobertura do rastreamento mamográfico informados na *Pesquisa Nacional de Saúde* (PNS) de 2019. Fonte: Departamento de Informática do SUS ^52^.

A comparação entre a produção total de mamografias de rastreamento e a necessidade para atender às mulheres de 50 a 69 anos indica que, caso toda a oferta fosse direcionada à população-alvo, o déficit de mamografias poderia ser reduzido a -14,8% no Brasil, enquanto na Região Sul haveria sobreoferta de 6,2% (Figura 1).

**Figura 2:**
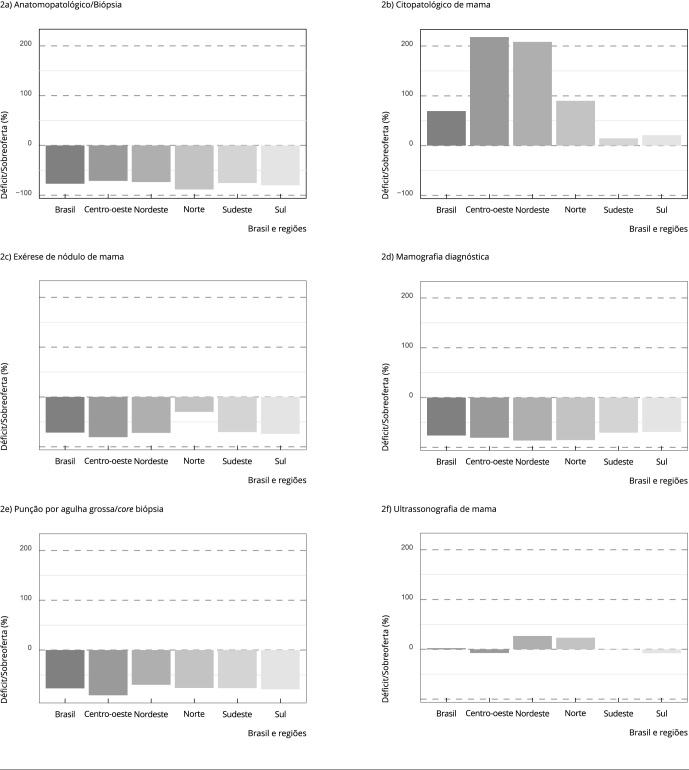
Déficit e sobreoferta de procedimentos de investigação diagnóstica para detecção precoce do câncer de mama feminino, estimados com base na produção registrada do Sistema Único de Saúde (SUS). Brasil e regiões, 2019.

Foram registradas no SIA/SUS, no ano de 2019, aproximadamente 209 mil mamografias diagnósticas e 33.500 PAG, indicando uma estimativa de déficit superior a 70% na realização de mamografias diagnósticas e PAG em todas as regiões do país. Os procedimentos apresentaram variações entre as regiões, sendo os maiores déficits de PAG (-90,8%) e biópsia/exérese de nódulo da mama (-80,6%) observados no Centro-oeste e o maior déficit de exames anatomopatológicos na região Norte (-88,5%) (Figura 2).

Fonte: Departamento de Informática do SUS ^52^.

Houve sobreoferta de exame citopatológico em todas as regiões do país, com valores mais altos no Nordeste (211,9%) e Centro-oeste (217,9%). A ultrassonografia mamária apresentou sobreoferta nas regiões Norte (23,3%), Nordeste (26,8%) e Sudeste (0,5%) (Figura 2).

## Discussão

Este estudo comparou a necessidade de procedimentos para a detecção precoce do câncer de mama no Brasil e a respectiva produção informada no SUS em 2019, revelando expressivo déficit na maioria dos procedimentos, com exceção de superávit de mamografia de rastreamento na Região Sul, de exame citopatológico de mama em todas as regiões e de ultrassonografia mamária nas regiões Nordeste e Norte.

O menor número de mulheres sintomáticas estimado para as regiões Norte e Nordeste, comparado ao Sudeste e Sul, explica-se pela distribuição desigual de fatores de risco no país, em parte relacionados ao padrão demográfico e à vida reprodutiva das mulheres, que diferem entre as regiões ^22^. É fundamental ter uma estimativa do número de mulheres com queixas mamárias em cada território, a fim de que a rede assistencial possa se preparar para garantir a elas atenção oportuna, já que o risco de confirmação de câncer de mama é maior em mulheres sintomáticas do que nas rastreadas ^4^. Destaca-se que, nas últimas décadas, o prognóstico de câncer de mama localmente avançado tem melhorado em virtude de avanços na terapia adjuvante ^23^, o que reforça a necessidade de acesso a diagnóstico e tratamento em tempo oportuno.

A necessidade de procedimentos para mulheres assintomáticas, de 50 a 69 anos, também deve ser prevista e baseada em evidências científicas ^4^ e no direcionamento mais adequado dos recursos ^24^. Os dados mostram que o déficit na oferta de mamografia de rastreamento diminuiria no país e em todas as regiões, e passaria a haver sobreoferta no Sul, se esses recursos fossem direcionados para a faixa etária alvo. A proporção de mamografias de rastreamento na população-alvo vem crescendo no Brasil, mas ainda é em torno de 65% ^25^, o que exige estratégias continuadas para capacitação profissional e adesão às diretrizes ^26^, cuja rejeição também causa o sobrerastreamento ^27^, impactando na oferta para a população-alvo.

A baixa cobertura do rastreamento mamográfico brasileiro e as discrepâncias entre as regiões Sul e Norte vêm sendo reportadas em alguns estudos ^28^^,^^29^^,^^30^, entretanto, o patamar apresentado na PNS (58,3%) está em nível intermediário quando comparado a coberturas de programas organizados em alguns países ^31^^,^^32^. Ressalta-se que a PNS se refere ao conjunto da população feminina, e não apenas às usuárias do SUS, e não distingue mamografia de “rastreamento” e “diagnóstica”, o que pode superestimar a cobertura nacional.

Os déficits superiores a 70% de mamografia diagnóstica, PAG e exame anatomopatológico, em todas as regiões do Brasil, expressam a dificuldade de acesso a recursos fundamentais para a confirmação diagnóstica no SUS, o que vem gerando grande atraso no diagnóstico, com consequente retardo do tempo para início do tratamento ^33^^,^^34^^,^^35^, reduzindo a sua efetividade ^36^. Os déficits mais expressivos de PAG (-90,8%) e biópsia/exérese de nódulo da mama (-80,6%), no Centro-oeste, e de exames anatomopatológicos, na Região Norte (-88,5%), reforçam lacunas já bem conhecidas no campo oncológico, cujos recursos tendem a ser ainda menores em regiões menos desenvolvidas ^15^^,^^37^^,^^38^. Segundo a Organização Mundial da Saúde (OMS) ^39^, a falta de investimento e a consequente infraestrutura inadequada de países de baixa e média renda para a oferta de serviços de diagnóstico de câncer, como os de patologia, resultam em serviços fragmentados que carecem de controle de qualidade.

O acesso limitado aos serviços de média complexidade no Brasil é um dos entraves na efetivação do princípio da integralidade no SUS. A tendência histórica de planejar as políticas de saúde na lógica da oferta instalada e não nas características epidemiológicas e necessidades sanitárias da população ^40^ acarreta demanda desarticulada da oferta ^41^, aumentando a busca por serviços privados e tensão entre fluxos formais e informais ^40^. Estudo com gestores da Bahia aponta que as dificuldades para a prestação de serviços especializados resultam da falta de integração e pactuação dos municípios e conflitos na relação público-privada ^41^.

A sobreoferta de exame citopatológico de mama em todas as regiões pode se justificar exatamente pela oferta reduzida de PAG no SUS e a tentativa de suprir, de alguma forma, esse procedimento recomendado na investigação de lesões mamárias suspeitas. Em algumas regiões, a sobreoferta superior a 100% é explicada por uma possível subestimação da necessidade de exames. Vale destacar que os parâmetros utilizados como referência ^14^ consideraram apenas a utilização do procedimento para pessoas sintomáticas com descarga papilar, porém o mesmo pode ser utilizado em outras indicações, como a investigação de cistos simples mamários. O exame citopatológico, embora não seja o padrão ouro para a confirmação do câncer, pode ser útil para investigar lesões palpáveis quando a PAG não estiver disponível, conforme a indicação clínica ^7^. Já no Centro-oeste, o excesso de ultrassonografias pode ser explicado por sua utilização inadequada como método de rastreamento, frequentemente solicitada em conjunto com a mamografia mesmo com recomendação contrária ^42^^,^^43^.

O cenário assistencial aqui apontado, marcado por carências de recursos para a detecção precoce, contribui decisivamente para manter elevado o patamar de apresentação avançada do câncer de mama. No Brasil, em 2020, cerca de 43% dos casos foram diagnosticados em estádios avançados ^10^, característica comum aos países de baixa e média renda com limitada capacidade diagnóstica. Reforçando as disparidades regionais, Renna Junior & Silva ^11^ constataram que, entre 2000 e 2012, a proporção de casos diagnosticados em estadiamento avançado variou no Brasil de 35% a 45% e que a chance de ter um diagnóstico em estágio avançado era 23% superior em mulheres tratadas na Região Norte e 61% naquelas tratadas no Centro-oeste quando comparadas às da Região Sul. As taxas de mortalidade são, consequentemente, diferentes, sobretudo quando se comparam capitais e interior, observando-se aumento no interior do Norte e Nordeste ^44^, apesar dessas regiões apresentarem menor número de casos de câncer de mama.

Um balanço crítico a ser feito é que, se o total de procedimentos de investigação diagnóstica realizados no Brasil, em 2019 - sem a indicação clínica discriminada por não estar disponível no SIA/SUS -, fosse direcionado apenas às mulheres sintomáticas, ainda assim seria insuficiente. É necessário fazer uma reflexão sobre a oferta responsável do rastreamento, com garantia da continuidade do cuidado, e do caráter de urgência e prioridade dos casos sintomáticos. Estratégias de organização dos serviços para maior agilidade na investigação e confirmação diagnóstica dos casos sintomáticos, e posterior organização do rastreamento, merecem ser debatidas.

De acordo com a OMS ^39^, o diagnóstico precoce do câncer depende da disponibilidade de recursos humanos e de tecnologias apropriadas, e os seus principais problemas são: fragilidades da atenção primária à saúde; inconsistência nos critérios para encaminhamento; falta de serviços de patologia; diagnóstico por imagem; e a distância geográfica de instalações que ofertam os procedimentos para o diagnóstico e o tratamento.

O rastreamento depende igualmente de infraestrutura necessária para diagnóstico e tratamento e de sistemas de informação para monitoramento de seus resultados ^45^. Para ser efetivo, é necessário cobrir proporção substancial da população alvo e assegurar o acesso ao diagnóstico e ao tratamento oportuno dos casos detectados. Todas as etapas, desde a oferta do rastreio até o tratamento, são essenciais para proporcionar os benefícios dessa estratégia e, assim, reduzir a ineficiência, os custos e os danos às mulheres ^39^.

O rastreamento organizado pressupõe: maior adesão às diretrizes (periodicidade e faixa etária), melhorando a eficiência, a efetividade e o balanço entre riscos e benefícios; implementação de controle de qualidade dos exames, com melhoria da acurácia da mamografia; e monitoramento dos casos alterados, diminuindo as perdas de seguimento. Vale ressaltar que o rastreamento organizado tende a ter menos casos de câncer de intervalo entre suas rodadas ^46^ e a ser mais eficiente do que oportunístico, ainda que a população-alvo seja a mesma ^47^.

O caráter oportunístico do rastreamento no Brasil dificulta o seu gerenciamento e sua adequação aos pressupostos de efetividade, entre os quais está a realização de mamografias na faixa etária recomendada. Um exemplo do gerenciamento inadequado das ações e recursos destinados ao rastreamento é a constatação deste estudo de que a Região Sul seria capaz de rastrear 58,3% das mulheres de 50 a 69 anos, conforme estimado pela PNS, se todas as mamografias de rastreamento realizadas no SUS fossem direcionadas à população-alvo. Apesar de ser um valor abaixo de 70%, a região, que apresenta a segunda maior incidência de câncer de mama do país, é a que tem maior capacidade de adequar sua oferta de mamografia de rastreamento às necessidades da população. Em nenhuma outra, ainda que todos os exames fossem direcionados à população alvo, seria possível atingir uma cobertura no SUS em consonância com o estimado pela PNS para o conjunto das mulheres brasileiras. Reforça-se que qualquer aumento de rastreamento deve ser acompanhado da previsão de serviços em toda a linha de cuidado para evitar gargalos assistenciais.

Diante das orientações de priorização de mulheres sintomáticas e dos pressupostos para implantação de um programa de rastreamento organizado, este estudo mostra que, no atual momento, o país precisa ampliar a capacidade diagnóstica para assegurar a realização de biópsias mamárias e de exames anatomopatológicos de forma a atender a todas as mulheres que deles necessitem. A ampliação da oferta desses procedimentos deve vir acompanhada de avaliação da suficiência e da demanda de capacitação dos profissionais envolvidos, com qualificação dos serviços e fluxos bem estabelecidos para aumentar o rendimento das unidades e, potencialmente, aumentar o acesso ^48^. É crucial definir estratégias para formar e fixar profissionais para a realização do exame histopatológico ^40^, recurso mais escasso no SUS. A organização da rede precisa ser revista à luz das demandas advindas dos territórios, com ampliação de acesso aos serviços especializados e busca de equidade e integralidade do cuidado, partindo-se da priorização da investigação diagnóstica de mulheres sintomáticas.

Cabe, por fim, alertar para que políticas que buscam incentivar o alcance de indicadores específicos, como o “aumento do rastreamento mamográfico” no contexto de pactuação de metas ^17^, não percam de vista a importância de organizar o conjunto da detecção precoce. Metas desarticuladas do seguimento das mulheres manterão o quadro preocupante aqui reportado.

Como limitação do estudo, aponta-se que os dados de cobertura de mamografia obtidos da PNS, ao se referirem a toda a população feminina, e não apenas às usuárias do SUS, podem ter superestimado a necessidade de mamografias de rastreamento, especialmente nas regiões Sul e Sudeste, que apresentam elevadas coberturas de saúde suplementar ^49^. Procurou-se minimizar essa tendência ao estimar a população possivelmente atendida no SUS.

A utilização da PNS para estimar a população rastreada no SUS pode ter, também, superestimado a cobertura de rastreio pela não distinção entre mamografias diagnósticas e de rastreamento. Acredita-se que essa tendência seja pequena para a estimativa de mamografias de rastreamento, visto que a maioria das mamografias é, de fato, realizada em mulheres assintomáticas. Conforme registrado no Sistema de Informação do Câncer (SISCAN), em 2021, 97,7% (2,63 milhões) das mamografias realizadas foram de rastreamento ^25^. Para a estimativa dos demais procedimentos, contudo, uma possível superestimação pode ter contribuído para acentuar um pouco a insuficiência de procedimentos da linha de cuidado do câncer de mama, no patamar aqui considerado.

Outro limite a ser considerado é a utilização de dados secundários para a análise da produção informada. Além de problemas relacionados à qualidade do dado, a ausência ou sub-registro dos procedimentos no SIA/SUS pode ter aumentado o déficit de oferta observado. O SIA/SUS é um sistema originalmente criado para faturamento e em algumas situações, quando a produção excede o teto financeiro pactuado, os procedimentos não são registrados ^50^, ficando sem informação.

A força deste estudo é oferecer um panorama mais completo e original da necessidade/oferta de procedimentos para a detecção precoce do câncer de mama no Brasil, incluindo não apenas mulheres assintomáticas, mas também sintomáticas. Esse conhecimento pode nortear o planejamento em saúde e a adoção de medidas mais assertivas para correção dos nós críticos identificados.

## Conclusão

A comparação entre a oferta e a necessidade de procedimentos para a detecção precoce do câncer de mama no Brasil e suas regiões identificou déficits e inadequações que devem ser melhor conhecidos e enfrentados em nível estadual e municipal.

Ao utilizar os parâmetros, considerando as diferenças regionais na incidência do câncer de mama no Brasil, foi possível mostrar um déficit generalizado de procedimentos, ao lado de especificidades regionais, como a sobreoferta de procedimentos de ultrassonografia mamária e de exames citopatológicos, nas regiões Centro-oeste, Norte e Nordeste.

Espera-se que os dados aqui apresentados contribuam para avançar o debate sobre a urgência de programar e adequar a rede assistencial, equacionando as necessidades do diagnóstico precoce e do rastreamento, visando ao uso mais eficiente e efetivo dos recursos na linha de cuidado do câncer de mama.
